# Reduced myocardial work in asymptomatic heavy alcohol use and its correlation with epicardial adipose tissue volume and serum biomarkers

**DOI:** 10.1002/clc.24151

**Published:** 2023-09-14

**Authors:** Canran Gao, Jie Yin, Tingting Hu, Shuai Liu, Xihai Zhao, Haiyan Ding, Xue Lin, Ligang Fang

**Affiliations:** ^1^ Department of Cardiology, State Key Laboratory of Complex Severe and Rare Diseases, Peking Union Medical College Hospital Chinese Academy of Medical Science and Peking Union Medical College Beijing China; ^2^ Department of Biomedical Engineering, Center for Biomedical Imaging Research Tsinghua University School of Medicine Beijing China

**Keywords:** alcohol, alcoholic cardiomyopathy, echocardiography, myocardial work

## Abstract

**Background:**

It is unclear whether long‐term heavy alcohol use leads to early cardiac function decline.

**Hypothesis:**

Long‐term heavy alcohol use developed reduced cardiac function in subclinical status by analyzing myocardial work (MW). Epicardial adipose tissue (EAT) volume and serum biomarkers contribute to identify potential factors sensitive in predicting early cardiac function decline.

**Methods:**

We enrolled 31 asymptomatic participants with heavy alcohol use and 33 age and sex‐matching nondrinking individuals. Participants underwent echocardiography, MW analysis, EAT volume measurement, serum biochemical examinations, and body composition assessment. We used multivariate linear regression to identify correlation between MW and total cholesterol (TC), EAT volume, and placental growth factor (PlGF). To determine global work efficiency (GWE) below the normal reference value of 96%, we developed receiver operating curves with area under curve (AUC) to compare different combinations of TC, EAT volume, and PlGF.

**Results:**

All 64 participants were male. GWE was reduced in the alcohol use group compared with the control group (96, interquartile range [IQR] = [95–97.75] vs. 97, IQR = [97–98], *p* = .004). TC was positively associated with GWE (*β* = .434, 95% confidence interval [CI] = 0.228 to 1.328, *p* = .008), whereas EAT volume (*β* = −.398, 95% CI = −0.000446 to −0.000093, *p* = .005) and PlGF (*β* = −.493, 95% CI = −1.010 to −0.230, *p* = .004) were inversely associated with GWE. The most significant AUC for reduced GWE was TC + EAT volume (0.851, 95% CI = 0.671 to 1, *p* = .006).

**Conclusion:**

Asymptomatic heavy alcohol use has shown early reduced cardiac function which can be associated with altered fat metabolism, suggesting individuals with alcohol use and abnormal fat metabolism need to be alert to heart damage.

## Graphical abstract

This cross‐sectional study showed that participants with heavy alcohol use but no cardiovascular disease symtoms experienced lower GWE than the control group, and the GWE correlated with TC, EAT volume, and PlGF. TC combined with EAT volume had a satisfactory prediction effect for GWE under 96% among all participants.

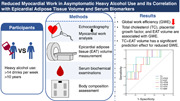

## 1 INTRODUCTION

Alcohol abuse is a major cause of nonischemic dilated cardiomyopathy which can eventually lead to heart failure or sudden cardiac death.^1^, ^2^ The latest nationwide cohort study revealed that 35.1% of Chinese adults are using alcohol,^3^ similar to 32.5% of the world population reported in 2011.^4^ Alcohol may directly induce toxicity in cardiomyocytes or indirectly damage in cardiovascular system. It has previously been observed that many patients with alcoholic cardiomyopathy (ACM) remain asymptomatic until they are diagnosed after presenting with symptoms of heart failure. Moreover, only a few individuals who die suddenly due to ACM have been previously diagnosed with cardiac disease.^2^ Given the increasing mortality and disability‐adjusted life years associated with ACM worldwide, there is a critical need for early screening of ACM in individuals with long‐term heavy alcohol use.^1^

We speculate that the effect of alcohol on the heart may be partly related to lipid metabolism and subepicardial fat content.^5^, ^6^ Therefore, in this study, we used various methods to test our speculation. Echocardiography was the main imaging technique used by past clinical research investigating alcohol impacts on the heart. However, conventional echocardiography is less sensitive than myocardial work (MW) analysis in detecting early cardiac function changes.^7^ MW analysis is a new noninvasive method for assessing cardiac function calculated using global longitudinal strain and left ventricular peak systolic pressure. Previously, MW analysis has been applied in studies on hypertension,^8^ coronary artery disease,^9^ heart failure,^10^ and cardiac resynchronization.^11^

In this study, we used MW analysis to evaluate cardiac function among asymptomatic participants with long‐term heavy alcohol use. Moreover, we applied various clinical examinations to find parameters correlated with early cardiac function change as detected by MW analysis, including epicardial adipose tissue (EAT) volume measurement, serum biochemical examinations, and body composition assessment. Total cholesterol (TC), EAT volume, and placental growth factor (PlGF) in the alcohol use group were examined in the study as potential independent variables to predict the global work efficiency (GWE) below the normal reference value of 96%.

The first aim of this study was to figure out whether asymptomatic heavy drinkers developed reduced cardiac function in subclinical status by analyzing MW. The second aim was to identify potential factors sensitive in predicting early cardiac function decline in individuals with heavy alcohol use.

## 2 METHODS

### 2.1 Study population

Participants in this study were recruited from residents who live near a distillery in Hebei Province in 2020. Recruitment information was shared through telephone communication. Inclusion criteria required participants aged over 18 with heavy alcohol use for more than 10 years, but with no cardiovascular disease symptoms. Heavy alcohol use was defined as over 14 drinks per week.^12^ A standard drink contained about 14 g of pure alcohol according to the US National Institute on Alcohol Abuse and Alcoholism.^13^ Participants who had a history of coronary artery disease, hypertension, diabetes, peripheral arterial disease, arrhythmia, renal diseases, cardiomyopathy, and liver cirrhosis were excluded. A total of 31 participants from 250 volunteers were screened as eligible. While no sex was preferred, all 31 alcohol drinking individuals recruited were male. The control group included 33 age and sex‐matched volunteers without alcohol use. All participants underwent echocardiography, MW analysis, EAT volume measurement, serum biochemical examinations, and body composition assessment. This study was approved by The Ethics Committees of Peking Union Medical College Hospital and Tsinghua University, and signed informed consent forms were obtained from all participants.

### 2.2 Alcohol consumption

We collected personal alcohol use habits of participants in the alcohol use group using a questionnaire, which inquired about types of alcoholic beverages, weekly alcohol use frequency, and the amount of alcohol consumed each time. To calculate the amount of alcohol consumption (g per week), 100 mL of liquor, beer, and wine were assumed to contain 31.5, 9.46, and 3.94 g of pure alcohol, respectively, based on the average alcohol concentration of each type available in the market. Weekly pure alcohol intake was calculated as frequency (per week) × amounts (L) × pure alcohol (g/L). The result was presented as standard drinks per week.

### 2.3 Echocardiography

Echocardiographic examination was performed using Vivid E9 ultrasound system (GE Vingmed Ultrasound AS) with an M5Sc probe by an experienced practitioner. Left ventricular (LV) systolic function was assessed by LV ejection fraction (LVEF) using Biplane Simpson method and global longitudinal strain (GLS). GLS was reported as an absolute value. Cardiac remodeling was primarily assessed by LV mass index (LVMI) over 109 g/m^2^ or relative wall thickness (RWT) over 0.51. The Chinese male thresholds of LVMI and RWT were defined by the Echocardiographic Measurements in Normal Chinese Adults (EMINCA) study.^14^, ^15^ LVMI was calculated by dividing left ventricular mass by body surface area.

### 2.4 MW analysis

Global and each LV segment MW were measured by vendor‐specific software (EchoPac Version 203, GE Vingmed Ultrasound AS, Horten, Norway). An experienced sonographer who was blinded to the group of participants performed the analysis. The calculation of MW required LV longitudinal strain, peak systolic LV pressure, and setting valvular event times. Brachial artery systolic blood pressure by cuff method was assumed to be equal to peak systolic LV pressure. LV longitudinal strain was measured by speckle tracking echocardiography which needed 3 cardiac cycles each for 3, 4, and 2 chamber apical views with 60–80/s frame rates. Valvular event times including mitral valve closure, aortic valve opening, aortic valve closure, and mitral valve opening were set using the apical three‐chamber view. The software provided a personalized LV pressure‐strain loop for each subject. The segmental MW was shown in a bull's eye plot. Two global MW indices were calculated as an average of segmental values: Global constructive work (GCW) was the average work during shortening in systole, indicating the MW contributed to pumping blood; Global wasted work (GWW) was the average work during lengthening in systole and shortening in isovolumic relaxation, indicating the MW not contributed to pumping blood. Global work index (GWI) was the area of the LV pressure‐strain loop. GWE was calculated as GCW divided by the sum of GCW and GWW. If every LV segment shortens during systole, the GWE will be 100%.^7^

### 2.5 EAT volume

Cardiac magnetic resonance (CMR, Philips Achieva TX 3.0T MRI) was used to measure EAT volume. An experienced radiologist traced out the contour of EAT compartments between visceral pericardium and myocardium using software (MATLAB 2014) and calculated the volume of EAT. EAT volume index was calculated as EAT volume divided by body surface area (BSA).

### 2.6 Serum biochemical examinations

All participants received serum biochemical examinations. Parameters in this study included: TC, low density lipoprotein cholesterol (LDL‐C), triglyceride (TG), uric acid (UA), albumin, pre‐albumin, thyroid stimulating hormone (TSH), adiponectin, lipocalin2/NGAL, resistin, plasminogen activator inhibitor‐1 (PAI1), and leptin. HOMA‐IR and HOMA‐β were used to assess insulin resistance according to the formula: HOMA‐IR = fasting insulin × fasting blood glucose/22.5, HOMA‐β = 20 × fasting insulin/(fasting blood glucose‐3.5). PlGF and vascular endothelial growth factor A (VEGFA) were detected for angiogenesis. Chronic inflammation was evaluated by the level of high‐sensitivity C‐reactive protein (hsCRP) and interleukin‐6 (IL‐6). Renal function was calculated according to the four‐variable modification of diet in renal disease equation: glomerular filtration rate (GFR, mL/min/1.7m^2^) = 186 × [serum creatinine (μmol/L)/88.4] −1.154 × age (years) −0.203.

### 2.7 Body composition assessment

The participants underwent body composition analysis by using InBody S10 bioelectrical impedance analyzer (Model JMW140, Biospace Co, Ltd.). The body composition parameters included in the study were body mass index (BMI), visceral fat area (VFA, cm2), visceral fat mass (VFM, kg), and subcutaneous fat mass (SFM, kg).

### 2.8 Statistical analysis

The Shapiro–Wilk was used to test the normality distribution of raw and log‐transformed continuous variables. The central tendency and dispersion of normally distributed variables were described using the mean ± standard deviation, while nonnormally distributed variables were described using the median and interquartile range (IQR). Categorical variables were expressed as frequency and percentage. To test the existence of significant differences between the alcohol use group and the control group, continuous variables used unpaired Student *t*‐test or the Mann–Whitney *U*‐test, as appropriate. Categorical variables were compared using Chi‐square test or Fisher exact test.

The correlation between MW and EAT volume and serum biomarkers in asymptomatic heavy alcohol use was tested using Spearman correlation coefficient and multivariate linear regression. Since the sample size of the alcohol use group was 31, we decided to select only 1–3 independent variables based on significant Spearman coefficient (*p* < .1) and clinical interest to make the regression result reliable.

A predictive model was then developed to determine the GWE below the normal reference value of 96%, in which receiver operating curves (ROC) with area under curve (AUC) were developed to compare different combinations of selected independent variables including TC, EAT volume and PlGF. Statistical analyses were performed using IBM SPSS Statistics for Windows, Version 22 software (IBM Corporation). All tests were two‐sided and their level of significance was set as *p* < .05.

## 3 RESULTS

### 3.1 Participant characteristics

This study enrolled 31 participants with asymptomatic heavy alcohol use as the alcohol use group and 33 age and sex‐matched participants without alcohol use as the control group. The demographic characteristics of all participants are shown in Table 1. The median alcohol consumption amount was 33 drinks per week (IQR = [21–53]). The duration of alcohol consumption was 27 ± 10 years. It should be noted that the participants who met the inclusion criteria were all male. In the alcohol use group. BMI, SBP, DBP, VFA, VFM, and SFM were significantly higher and 18 out of 31 participants were in smoking status. Table S1 shows the comparison of serum biomarkers and EAT volume between the two groups. GFR, absolute value, hsCRP, PlGF, log VEGFA, log Lipocalin2NGAL, and log PAI1 were significantly higher and HOMA‐IR was significantly lower in the alcohol use group in comparison with the control group (*p* < .05).

**Table 1:** Demographic characteristics.

	Alcohol (*n* = 31)	Control (*n* = 33)	*p* value
Age (years)	50 ± 10	49 ± 10	.581
Gender	male		
BMI (kg/m^2^)	26.16 ± 3.49	23.58 ± 1.90	.001
Smoking	18 (62)	0 (0)	.000
Alcohol consumption (drinks/week)	33 (21–53)		
Duration of alcohol consumption (years)	27 ± 10		
SBP (mmHg)	122 ± 13	116 ± 10	.038
DBP (mmHg)	80 ± 8	73 ± 6	.000
HR (beats/min)	73 ± 7	71 ± 10	.401
VFA (cm^2^)	119.5 (105.25–130)	109 (98.5–116)	.010
VFM (kg)	2.66 ± 0.95	2.10 ± 0.51	.006
SFM (kg)	15.22 ± 4.18	12.82 ± 2.53	.008

Abbreviations: BMI, body mass index; DBP, diastolic blood pressure; HR, heart rate; SBP, systolic blood pressure; SFM, subcutaneous fat mass; VFA, visceral fat area; VFM, visceral fat mass.

### 3.2 Global MW and conventional echocardiography

The conventional measurement showed a significant difference in IVSd, LVPWd, LVM, LVMI, and RWT, indicating a tendency of cardiac remodeling in the alcohol use group.

MW analysis showed that GWE was reduced in the alcohol use group compared with the control group (96, IQR = [95–97.75] vs. 97, IQR = [97–98], *p* = .004). No significant differences were found in GWI, GCW, and GWW between the two groups (*p* > .05) (Table 2).

**Table 2:** Conventional echocardiographic parameters and global MW indices.

	Alcohol (*n* = 31)	Control (*n* = 33)	*p* value
IVSd (mm)	10 ± 2	9 ± 2	.002
LVIDd (mm)	47 ± 5	48 ± 4	.659
LVIDs (mm)	28 ± 5	28 ± 3	.394
LVPWd (mm)	10 (9–11)	9 (8–9)	.000
E (m/s)	0.71 ± 0.17	0.73 ± 0.20	.633
A (m/s)	0.66 ± 0.20	0.66 ± 0.21	.993
E/A ratio	1.18 (1–1.38)	1.23 (0.80–1.46)	.862
LVM (g)	175 ± 40	146 ± 32	.002
LVMI (g/m^2^)	93 (76–107)	80 (64–93)	.003
RWT	0.43 (0.35–0.51)	0.36 (0.31–0.39)	.002
LVEF (%)	65 ± 5	67 ± 5	.327
Cardiac remodeling	12 (38.7)	3 (9.1)	.007
GLS (%)	19.3 ± 2.0	20.2 ± 2.0	.064
GWI (mmHg%)	1794 (1574–2029)	1806 (1655–1884)	.680
GCW (mmHg%)	2057 ± 312	2024 ± 237	.634
GWW (mmHg%)	63 (35–82)	39 (28–50)	.060
GWE (%)	96 (95–97.75)	97 (97–98)	.004

Abbreviations: E/A ratio, the ratio of the early (E) to late (A) diastolic mitral valve blood flow velocities; GCW, global constructive work; GLS, global longitudinal strain; GWE, global work efficiency; GWI, global work index; GWW, global wasted work; IVSd, interventricular septum thickness at diastole; LVEF, left ventricular ejection fraction; LVIDd, left ventricualr internal dimension diastole; LVIDs, left ventricular internal dimension systole; LVM, left ventricular mass; LVMI, Left ventricular mass index; LVPWd, left ventricular posterior wall thickness; RWT, Relative wall thickness.

### 3.3 TC, EAT volume, and PlGF as covariates on GWE

Correlation and regression analysis was conducted to identify the biomarkers covariation with global MW in the alcohol use group (*n* = 31). The positive results of the correlational analysis were presented in Table 3. TC (*r* = −.364, *p* = .048) and LDL‐C (*r* = −.374, *p* = .042) showed negative correlations with GCW. HOMA‐β is negatively correlated with GWI (*r* = −.482, *p* = .007) and GCW (*r* = −.564, *p* = .001). No significant correlation was found in other clinical characteristics with global MW indices. Table S2 presented the full result of Spearman coefficient.

**Table 3:** Spearman correlation coefficient.

	GWI (mmHg%)		GCW (mmHg%)		GWW (mmHg%)		GWE (%)	
	*r*	*p* value	*r*	*p* value	*r*	*p* value	*r*	*p* value
TC (mmol/L)	−.329	.076	−.364*	.048	−.053	.782	.035	.852
LDL‐C (mmol/L)	−.303	.103	−.374*	.042	−.108	.568	.105	.580
HOMA‐β (%)	−.482**	.007	−.564**	.001	−.244	.194	.073	.702

*Note*: **p < .01. *p < .05.

Abbreviations: GCW, global constructive work; GWE, global work efficiency; GWI, global work index; GWW, global wasted work; HOMA‐β, homeostasis model assessment for β‐cell function; LDL‐C, low density lipoprotein cholesterol; TC, total cholesterol.

Because reduced GWE in the alcohol use group had statistical significance, we chose GWE as the dependent variable in the regression analysis. TC, EAT volume, and PlGF were selected as independent variables for the model. As shown in Table 4, TC was positively associated with GWE (*β* = .434, 95% confidence interval [CI] = 0.228 to 1.328, *p* = .008), whereas EAT volume (*β* = −.398, 95% CI = −0.000446 to −0.000093, *p* = .005) and PlGF (*β* = −.493, 95% CI = −1.010 to −0.230, *p* = .004) were inversely associated with GWE.

**Table 4:** Multivariate linear regression with GWE as the dependent variable.

Regression variable	*B*	*β*	95% CI	VIF	*p* value
Constant term	95.082				
TC	0.778	.434	0.228 to 1.328	1.429	.008
EAT volume	0.000	−.398	−0.000446 to −0.000093	1.036	.005
PlGF	−0.620	−.493	−1.010 to −0.230	1.462	.004

*Note*: *N* = 31. Adjusted *R* ^2^ = .669. *p < .05.

Abbreviations: CI, confidence interval; TC, total cholesterol; VIF, variance inflation factors.

### 3.4 Different independent variables combined with TC to predict reduced GWE

**Figure 1:**
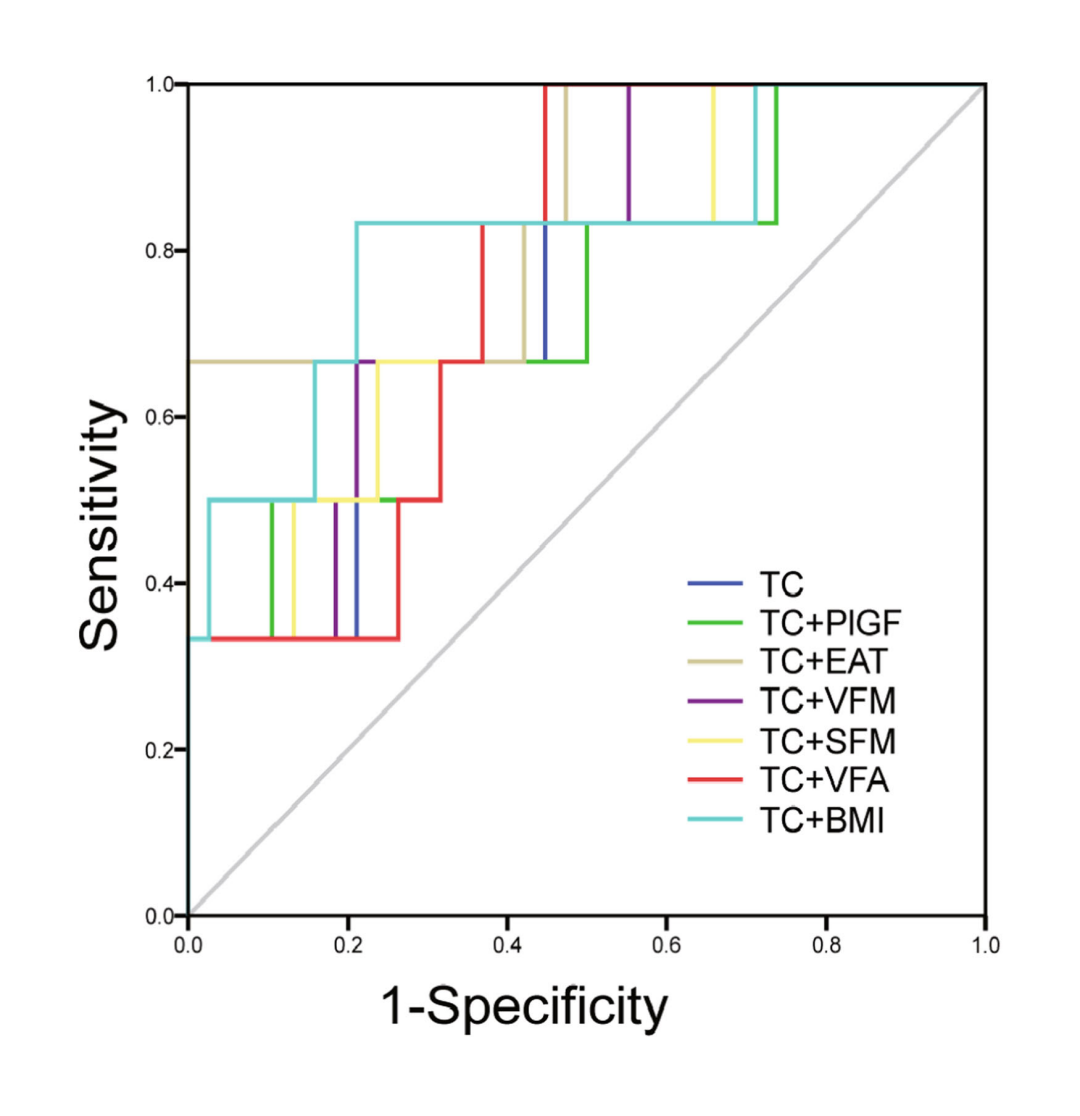
ROC curves for TC combined with different variables to predict reduced GWE. BMI, body mass index; EAT, epicardial adipose tissue; PlGF, placental growth factor; SFM, subcutaneous fat mass; TC, total cholesterol; VFA, visceral fat area; VFM, visceral fat mass.

We combined TC with covariates mentioned above and body composition parameters including BMI, VFM, SFM, and VFA to test the prediction effect of different combinations for reduced GWE. Figure 1 shows the ROC curves of TC + EAT volume, TC + PlGF, TC + VFM, TC + SFM, TC + VFA, TC + BMI, and TC for reduced GWE in all participants (*n* = 64). AUC for all combinations was presented in Table S3. The most significant AUC for reduced GWE was TC + EAT volume (0.851, 95% CI = 0.671 to 1, *p* = .006). Another interesting result was that the AUC of TC + BMI (0.816, 95% CI = 0.607 to 1, *p* = .014) showed no significant difference with TC + EAT volume (*p* = .6528). Table S3 suggested a further pairwise comparison of ROC curves using DeLong's test. No significant difference between either pair was evident.

## 4 DISCUSSION

This cross‐sectional study showed that participants with heavy alcohol use but no cardiovascular disease symtoms experienced lower GWE than age and sex‐matched control group, and the GWE correlated with TC, EAT volume, and PlGF. TC combined with EAT volume had a satisfactory prediction effect for GWE under 96% among all participants while TC combined with BMI also showed a statistically similar result.

Our study confirms that fat metabolism may be associated with altered cardiac function in heavy alcohol use. Recently, fat is regarded as an active endocrine organ that could profoundly influence metabolism.^16^ In this study, we found that the target heavy alcohol use group had more overweight participants categorized by BMI and higher fat depots by body composition assessment.

EAT is fat depots adjoining the myocardium without a barrier, allowing for more convenient cell communication between EAT and myocardium. EAT covers 80% of the heart surface, mainly in right ventricle surface, anterior wall of the left ventricle, and anterior and lateral walls of right atrium. EAT is composed of adipocytes, lymphocytes, and macrophages.^17^ In healthy people, EAT is involved in thermoregulation and free fatty acid storage for cardiomyocytes.^18^ In obesity or other metabolic disorder situations, normal adipocytes grow into hypertrophy adipocytes followed by adipose tissue volume expansion Blood perfusion cannot meet the oxygen consumption requirements of the expanded EAT, resulting in an ischemic environment. Hypertrophic adipocytes secrete hypoxia inducible factor‐1α to promote collagen production and activate myocardial fibrosis,^19^, ^20^ which is an important pathological manifestation of adverse myocardial remodeling. In a previous study on atrial fibrillation, hypertrophic adipocytes and activated macrophages secreted tumor necrosis factor‐α and interleukin‐6 (IL‐6) to activate NOD‐like receptor family protein‐3 (NLRP3) inflammatory pathway and promote atrial fibrosis and electrical remodeling.^21^, ^22^, ^23^ EAT in rat myocardial infarction model expanded the surface area of H9C2 cells and activated cardiac fibroblasts into myofibroblasts by increasing the level of reactive oxygen species in cardiac fibroblasts. The study suggested a novel pathological mechanism of cardiac remodeling by EAT upregulating miR‐134‐5p in target cells.^24^ Previous studies showed that EAT volume was significantly higher in HFpEF patients and EAT volume had a negative correlation with left ventricular diastolic relaxation and filling.^25^ Our study suggests that the relationship between EAT and cardiac remodeling in alcohol use deserves attention, especially the interaction between EAT and the myocardium, which may provide new ideas for studies on alcohol‐induced myocardial disease.

Interestingly, PlGF is related to GWE in this study. PlGF is a member of VEGF family synthesized from placental, playing an important role in vascular inflammation and adverse outcome in patients with acute coronary syndrome,^26^ metabolic syndrome,^27^ as well as childhood obesity.^28^ Recently increasing data also showed that the development and maintenance of fat depots require angiogenesis in which VEGF and its receptors play a crucial role.^29^, ^30^ For example, PlGF is a sensitive indicator of women with acute fatty liver during pregnancy.^31^ In sum, our study suggests that PlGF may be a key factor in alcohol‐induced cardiac dysfunction which is possibly related to its role in fat metabolism and angiogenesis.

MW analysis showed an earlier cardiac function change on GWE and an increasing tendency of wasted work in the alcohol use group. At the same time, the conventional echocardiography showed no significant EF change but the heart was in the progress of cardiac remodeling. This result indicated that MW analysis was a sensitive method to help individuals with heavy alcohol use but no cardiovascular disease symptoms to detect reduced cardiac function. For better screening of reduced cardiac function among the population, we did ROC curves of common clinical examination parameters to determine GWE under 96%. TC + EAT volume had the most significant AUC and TC + BMI also showed good prediction value. By comparing the AUC of TC + EAT volume and TC + BMI, we found that the two combinations have statistically the same prediction efficacy. Among the population, lower TC and elevated BMI imply reduced cardiac work efficiency, especially in long‐term heavy alcohol use. Prior studies have noted the importance of alcohol abstinence to prevent ACM advance.^32^, ^33^ Because alcohol use as a lifestyle is difficult to change, it is necessary to find indicators that can guide patients towards alcohol cessation at the appropriate time. The GWE provided by our study may be a reference indicator.

This study has several limitations: First, this research had a limited sample size for both the alcohol use and the control groups. Only 31 participants met the inclusion criteria of the former from 250 volunteers. Second, the participants were all Chinese from northern China. There might be geographical bias and ethnic bias.^1^ Third, in this study, only male participants met the selection criteria and thus were included. The early heart function change in females remains unknown. Fourth, 62% participants in the alcohol use group were smokers. Previous study suggested the association between smoking and ventricular hypokinesis.^34^ Further exploration with larger sample size and subgroup analysis is needed to investigate whether smoking is a confounder in the effect of alcohol on myocardium.

## 5 CONCLUSION

The purpose of the current study was to determine reduced cardiac function in individuals with heavy alcohol use but no cardiovascular disease symptoms using MW analysis, and to find out which parameters commonly used in clinical examinations are sensitive in predicting early cardiac function decline. The study has shown that GWE is significantly reduced in heavy alcohol use and correlates with TC, EAT volume, and PlGF. Further ROC curves tested the prediction ability for reduced GWE by combining TC with different variables. TC + EAT volume and TC + BMI have significant AUC.

## CONFLICT OF INTEREST STATEMENT

The authors declare no conflicts of interest.

## Supporting information

Supporting information.

Supporting information.

Supporting information.

## Data Availability

The data that support the findings of this study are available from the corresponding author upon reasonable request.
